# Anxiolytic, Antidepression, and Memory-Enhancing Effects of the Novel Instant Soup RJ6601 in the Middle-Aged of Female Rats

**DOI:** 10.3390/foods13142170

**Published:** 2024-07-09

**Authors:** Rujikan Chaisanam, Jintanaporn Wattanathorn, Wipawee Thukham-mee, Nawanant Piyavhatkul, Pongsatorn Paholpak

**Affiliations:** 1 Department of Physiology and Graduate School (Neuroscience Program), Faculty of Medicine, Khon Kaen University, Khon Kaen 40002, Thailand; c_rujikan@kkumail.com; 2 Department of Physiology, Faculty of Medicine, Khon Kaen University, Khon Kaen 40002, Thailand; meewep@gmail.com; 3 Research Institute for High Human Performance and Health Promotion, Khon Kaen University, Khon Kaen 40002, Thailand; 4 Department Psychiatry, Faculty of Medicine, Khon Kaen University, Khon Kaen 40002, Thailand; nawanant@kku.ac.th (N.P.); ppaholpak@kku.ac.th (P.P.)

**Keywords:** polyphenol, dietary fiber, mental health, anxiolytic, antidepression, memory-enhancing effect

## Abstract

Due to the health benefits of polyphenols and dietary fiber in combating mental disorders, we hypothesized that a polyphenol- and dietary fiber-enriched soup (RJ6601) would improve mental wellness in a rat model of middle-aged women. To test this hypothesis, female Wistar rats aged 18 months (350–450 g) were orally administered RJ6601 at doses of 200 and 400 mg/kg BW for 28 days. The anxiolytic, antidepression, and memory-enhancing effects were assessed every 7 days throughout the study period. The neuron density and levels of activities of AChE, total MAO, MAO-A, MAO-B, MDA, SOD, CAT, GSH-Px, IL-1β, IL-6, and BDNF in the prefrontal cortex at the end of study were also investigated. Furthermore, the amounts of *Lactobacillus* spp. and *Bifidobacterium* spp. in their feces were also determined. The results revealed that the developed soup shows anxiolytic, antidepression, and memory-enhancing effects. An increased neuron density; reductions in AChE, total MAO, MAO-A, MAO-B, and MDA; and an elevation of serum BDNF, together with increased amounts of both bacterial species in feces, were also observed. Our results suggest that RJ6601 is a potential mental wellness promotion supplement that enhances BDNF levels, brain plasticity, neurotransmitter balance, and oxidative stress and inflammation status, along with improving microbiota.

## 1. Introduction

Numerous changes occur in mid-life, including social, biological, and psychological changes. These changes have a greater impact on the mental wellness of women than men [1]. The increasing risk of mental health disorders in middle-aged women is drawing attention to mental health problems [2]. Among the various mental health disorders, anxiety, depression, and dementia are regarded as the most common illnesses affecting women globally [3,4]. The global prevalence of anxiety is approximately 5.15–5.76% [5], whereas the global prevalence of depression is around 7.7–9.4% [6]. The prevalence rate of mild cognitive impairment in middle-aged women, which tends to arise during the menopausal transition period, is around 44–62% [7]. Therefore, mental disorders are responsible for the largest proportion of the global burden of disease and a leading cause of disability and poor quality of life worldwide [8,9]. Most of the current therapeutic strategies against the aforementioned conditions focus on individual disease, and no available strategy focuses on all conditions together, even though they can coexist. As a result of this situation, individuals who suffer from such conditions may take numerous drugs, which is not convenient. Owing to the great impacts of these diseases and the inconvenience experienced by affected individuals, adequate therapeutic and preventive strategies for mental health promotion are required.

There is a wealth of evidence demonstrating that both inflammation and oxidative stress play crucial roles in the pathogenesis and progression of dementia and emotional disorders such as anxiety and depression [10,11,12]. Mounting evidence is showing that they are linked to gut dysbiosis [13,14,15]. With the advancement of age, there is a decrease in the diversity of gut microbiota composition. *Proteobacteria* spp. appear to increase in abundance, whereas *Bifidobacteria* spp. decrease. This change gives rise to a reduction in short chain fatty acids (SCFAs) [16] that leads to the elevation of inflammatory cytokines and oxidative stress, which finally results in the disturbance of neuronal functions in the pathways involved in mood regulation and memory [17,18,19,20]. Owing to the crucial roles of gut microbiota dysbiosis, oxidative stress, and inflammation in the pathophysiology of mood disorders and memory impairment mentioned earlier, gut microbiota inflammation and oxidative stress have gained attention as preventive and therapeutic targets for modification in mental health intervention. The current available drugs still exhibit the potential for side effects such as agitation, Stevens–Johnson syndrome, toxic epidermal necrolysis, and neuroinflammation [21,22]. In addition to the current medications, both probiotics and symbiotics can also be used to modify the mentioned condition, but there is the potential for antibiotic-resistance genes to be transferred from probiotics to other gut microflora, eventuating in opportunistic pathogens sharing the same intestinal habitat, and this can induce serious clinical conditions [23]. Therefore, the soup investigated in this study is a potential candidate as a functional supplement for mood disorders and memory impairment with fewer serious side effects, such as the potential to transfer antibiotic-resistance genes.

Accumulating evidence over the past decade has demonstrated that polyphenolic compounds, which possess antioxidant and anti-inflammatory effects, can improve cognitive function and mitigate symptoms of psychiatric disorders [24,25,26]. They also modulate gut microbiota as well as the function of the gut–brain–axis, resulting in an alleviation of neuroinflammation, oxidative stress reduction, and serotonin metabolism balance [27], important factors that play critical roles in the pathophysiology of mood disorders and memory impairment. Communication in the gut–brain axis encompasses many pathways involving the vagal nerve, the hypothalamic–pituitary–adrenal axis (HPA-Axis) via cortisol secretion, the production of bacterial metabolites such as short chain fatty acids (SCFAs), immune mediators such as cytokines, and the neuroendocrine signals of hormones and neurotransmitters [28]. In addition to polyphenols, dietary fiber can also modulate inflammation and antioxidant effects. After consumption, dietary fiber cannot be digested and transits to the intestine. It is then fermented by the intestine microbiota to generate small metabolite molecules, for example, short chain fatty acids (SCFAs) such as butyrate, which exerts important cellular functions related to homeostasis, especially antioxidant and antioxidant activity [29]. Furthermore, these metabolites can also stimulate the gut–brain axis, giving rise to the improvement in mental health status [30,31,32]. Owing to the health benefits of polyphenols and dietary fiber, as mentioned earlier, we focus on the mental wellness promotion effect of a functional soup rich in polyphenols and dietary fiber in this study. We hypothesize that the consumption of a polyphenol- and dietary fiber-enriched soup should result in improvements related to anxiety, depression, and memory in an animal model of middle-aged women. Therefore, we designed this study to investigate this hypothesis and explore the possible underlying mechanisms.

## 2. Materials and Methods

### 2.1. Instant Soup “RJ6601” Formulation

**Figure 1:**
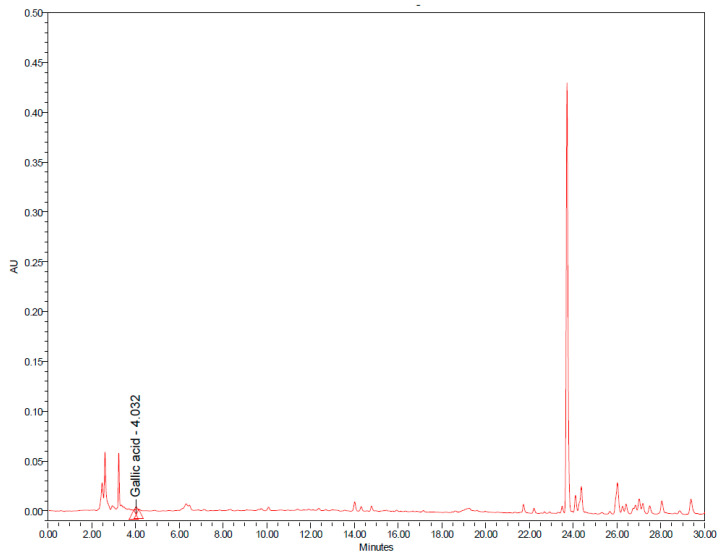
HPLC chromatograms of functional soup at a concentration of 500 mg/mL and wavelength of 275 nm.

**Figure 2:**
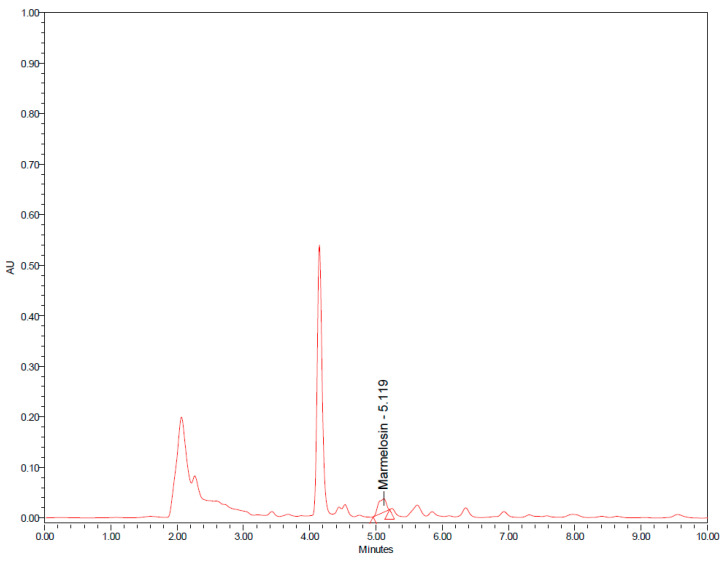
HPLC chromatograms of functional soup at a concentration of 500 mg/mL and wavelength of 247 nm.

RJ6601 is a novel soup that is rich in polyphenolic compounds and dietary fiber. This functional soup was prepared by mixing together the ingredients shown in Table 1, and then, a natural green colorant from kale and celery was added to the functional soup, whereas a green synthetic colorant was added to the placebo soup. Both types of soup contain 0.4% colorant. Furthermore, inulin was added to both the placebo soup and functional soup at a concentration of 5%. A serving of the functional soup provides around 4.21 ± 0.01 kcal/g in energy, whereas a serving of the placebo soup provides 4.35 ± 0.07 kcal/g in energy. RJ6601 contains dietary fiber and polyphenolic compounds at around 5.59 ± 0.04 g/g sample and 84.42 ± 0.02 mg GAE/g sample, whereas placebo soup contains these substances at concentrations of 1.12 ± 0.06 g/g sample and 64.42 ± 0.02 mg GAE/g sample. Furthermore, an analysis of the ingredients showed that it contains gallic acid, quercetin, and marmelosin at concentrations of 5 ± 0.000, 15 ± 0.000, and 11 ± 0.00 μg/g of sample powder. The fingerprint chromatogram of the developed soup was shown in Figure 1 and Figure 2.

### 2.2. Experimental Animals and Protocol

All the procedures involving experimental rats used in this study were conducted according to the Affairs Concerning Experimental Animals and approved by the Institutional Animal Care and Use Committee of Khon Kaen University, Thailand (Record no. IACUC-KKU-31/64). Female Wistar rats aged 18 months and weighing 350–450 g were obtained from the Northeast Laboratory Animal Center, Khon Kaen University, Khon Kaen province, Thailand. They were maintained under normal conditions without specific pathogens at 22 ± 2 °C on 12:12 h light/dark cycle. All rats were adaptively fed for a week, with four rats per cage, before starting the experiments. They were randomized and divided into various groups (N = 8/group) as described in Table 2.

The animals in Groups I–IV were administered the assigned substance for 4 weeks. The selected doses of RJ6601 used in this study were derived from transferring the EC50 of the surrogate markers related to the pathophysiology of the interested conditions according to the assumption that the rat body was a one-compartment model. The anxiolytic and antidepression effects were respectively assessed using an elevated plus maze and forced swimming tests, whereas the memory-enhancing effect was assessed using the Morris water maze and novel object recognition tests. All the behavioral assessments were performed every 7 days throughout a 28 day study period. At the end of study, they were assessed regarding oxidative stress status, including malondialdehyde (MDA) levels; the activities of superoxide dismutase (SOD), catalase (CAT), and glutathione peroxidase (GSH-Px) together with inflammatory markers such as interleukin-1β (IL-1β) and interleukin-6 (IL-6); and neurotransmitter changes via the measurement of acetylcholinesterase (AChE), total monoamine oxidase (MAO), monoamine oxidase type A (MAO-A), and monoamine oxidase type B (MAO-B) in the cerebral cortex. Serum brain-derived neurotrophic factor (BDNF) was also investigated. Furthermore, the amounts of *Lactobacillus* spp. and *Bifidobacterium* spp. in feces were also characterized. For this last part, which was set up to explore the possible mechanism, no positive control was used because no gold standard supplement was available.

### 2.3. Behavioral Assessments

#### 2.3.1. Elevated plus Maze Test

In this study, the anxiolytic effect was assessed using an elevated plus maze test, an extensively validated animal model of anxiety that capitalizes on the natural aversion of rodents for open spaces and the elevated structure of the maze [36,37,38]. The plus maze consisted of two open arms (50 × 10 cm) and two enclosed arms (50 × 10 cm) with walls of 40 cm height, elevated 50 cm above the ground. The rats were tested on the maze in randomized order. The test was initiated by placing each rat in the center of the maze, facing one of the open arms, and letting it move freely. Each test lasted 5 min, and the performance of each rat in the test was recorded using a video camera. The maze was thoroughly cleaned between each test. The number of entries into open arms and the time spent in the open arms were recorded and used as an indicator of anxiolytic activity. This test was performed before the intervention, after a single dose of substance administration, and every 7 days throughout the 28 day experimental period.

#### 2.3.2. Forced Swimming Test

Antidepression-like activity of the soup was assessed using the forced swimming test, the most frequently used animal model for assessing antidepressant behavior. Rats were placed in a glass cylinder tank filled with water at 25 °C, and the level of water was sufficiently high such that neither their paws nor their nails touched the bottom of the tank. Each rat was exposed to a 6 min test. The test was performed at baseline, after a single dose of test substance administration, and on days 7, 21, and 28 after the administration of the tested substances. All the performances were recorded using a video camera and analyzed for immobility, swimming, and climbing times [39].

#### 2.3.3. Morris Water Maze Test

In this study, hippocampal-dependent spatial memory was monitored using a Morris water maze test, the details of which are mentioned elsewhere [35,40]. In brief, each rat was trained to memorize both its location in the four-quadrant circular pool and the location of the immersed platform in one of the mentioned pools by using the external cues in the surrounding area. After 5 days of trials (two trials/day), each rat was exposed to the test session. Each test consisted of two phases: acquisition and probe trial. In the acquisition phase, escape latency, or time spent finding the immersed platform, was monitored, whereas the retention time was measured in the probe trial phase, which was determined 24 h after the assessment of acquisition phase—in this trial, the immersed platform was removed from the test. The assessment was performed prior to the intervention and after the intervention at various time points, including after a single administration event and on days 7, 14, 21, and 28.

#### 2.3.4. Novel Object Recognition Test

In this study, a novel object recognition (NOR) assay, a sensitive procedure for evaluating compounds for cognition-enhancing activity, was applied to determine the effects of the functional soup on nonspatial memory. All experimental rats were acclimatized in an open-field area in the absence of objects for 30 min, one day before the NOR test was performed. Then, each rat was acclimatized again before testing for 3 min and exposed to two identical objects (A and B, with similar size, shape, and color) which were in a separated area in an open field. Then, the animal was allowed to explore the objects for 3 min and returned to its home cage for 15 min. Then, it was re-exposed to an open field in which both the familiar (A) and novel (C) objects were located, and each animal was allowed 3 min of exploration time. Exploration duration, which was defined as the time that the rats spent exploring the objects by sniffing or touching within 2 cm, was recorded and calculated for determining the discrimination index [41] according to the equation below. The assessment was carried out at 30, 60, and 120 min after the administration of the tested substances.

Discrimination Index = [TC − TA/(TA + TC)] × 100

TA—total duration of exploration with object A in testing session; TC—total duration of exploration with object C in testing session.

### 2.4. Biochemical Assessment

#### 2.4.1. Measurement of Oxidative Stress Markers

After sacrifice at the end of the study, the brains were removed and homogenized with 50 volumes of 0.1 M ice-cold phosphate buffer saline. The homogenate was subjected to 3000× *g* centrifugation at 4 °C for 15 min. Then, the supernatant was collected and used for bioassays. The current study involved monitoring a metabolic product of lipid peroxidation (LPO) or malondialdehyde (MDA) and the primary endogenous antioxidant enzymes, including superoxide dismutase (SOD), catalase (CAT), and glutathione peroxidase (GSH-Px), which were used as indicators of the oxidative stress status of the brain tissue.

MDA levels were assessed using a thiobarbituric acid (TBA) test. Briefly, 50 μL of brain tissue homogenate was mixed with a reaction mixture containing 50 μL of 8.1% sodium dodecyl sulphate (SDS) (Sigma-Aldrich, St. Louis, MO, USA), 375 μL of 0.8% thiobarbituric acid (TBA) (Sigma-Aldrich, St. Louis, MO, USA), 375 μL of 20% acetic acid (Sigma-Aldrich, St. Louis, MO, USA), and 150 μL of distilled water (DW). Following this step, the solution was heated at 95 °C in a water bath for 60 min. At the end of incubation, it was cooled with tap water and mixed with 250 μL of distilled water and 1250 μL of an n-butanol and pyridine solution (15:1; Merck, Darmstadt, Germany). Then, it was subjected to 4000 rpm for 10 min, and the upper layer was collected for determination of absorbance at 532 nm. TMP (1,1,3,3-tetramethoxy propane) was used at concentrations ranging 0–15 μM (Sigma-Aldrich, St. Louis, MO, USA) for the preparation of a standard calibration curve. MDA levels are expressed as ng/mg protein [42].

SOD activity was measured based on the suppression of the nitroblue tetrazolium (4-nitroblue tetrazolium chloride, NBT) reduction rate in the nonenzymatic phenazine methosulfate–nicotinamide adenine dinucleotide system, as previously described [36]. In brief, 200 μL of a reaction mixture containing 57 mM of phosphate buffer solution (KH_2_PO_4_) (Sigma-Aldrich, St. Louis, MO, USA), 0.1 mM of EDTA (Sigma-Aldrich, St. Louis, MO, USA), 10 mM of cytochrome C (Sigma-Aldrich, St. Louis, MO, USA) solution, 50 μM of xanthine (Sigma-Aldrich, St. Louis, MO, USA), and 20 μL of xanthine oxidase (0.90 mU/mL) (Sigma-Aldrich, St. Louis, MO, USA) was mixed with 20 μL of the brain tissue homogenate. Then, absorbance at 415 nm was recorded. The standard calibration curve was prepared using the SOD enzyme (Sigma-Aldrich, St. Louis, MO, USA) at concentrations of 0–25 units/mL, and the results are expressed as units/mg protein.

The measurement of CAT activity was performed according to the method of Palachai et al. [42]. An assessment was carried out based on the degradation of H_2_O_2_ by CAT. Briefly, an aliquot of brain tissue at a volume of 10 μL was mixed with a reaction mixture consisting of 50 μL of 30 mM hydrogen peroxide (in 50 mM phosphate buffer, pH 7.0) (BDH Chemicals Ltd., London, UK), 25 μL of 4 M H_2_SO_4_ (Sigma-Aldrich, St. Louis, MO, USA), and 150 μL of 5 mM KMnO_4_ (Sigma-Aldrich, St. Louis, MO, USA). Then, the absorbance at 490 nm was measured.

GSH-Px activity assessment was performed using the method of Palachai et al. [42]. An assay mixture consisting of 10 μL of 1 mM dithiothreitol (DTT) (Sigma-Aldrich, St. Louis, MO, USA) in 6.67 mM of potassium phosphate buffer (pH 7), 10 μL of 50 mM glutathione solution (Sigma-Aldrich, St. Louis, MO, USA), and 100 μL of 30% hydrogen peroxide (BDH Chemicals Ltd., London, UK). After a 5 min incubation period, 10 μL of 10 mM 5,5-dithiobis-2-nitrobenzoic acid (DTNB) (Sigma-Aldrich, St. Louis, MO, USA) was mixed with 20 μL of brain sample and incubated for 5 min. Then, 10 μL of 10 mM 5,5-dithiobis-2-nitrobenzoic acid (DTNB) (Sigma-Aldrich, St. Louis, MO, USA) was added by shaking for 5 min, and mixed with 10 μL of 10 mM DTNB (5,5-dithiobis-2-nitrobenzoic acid) (Sigma-Aldrich, St. Louis, MO, USA), and the absorbance at 412 nm was determined using a microplate reader. GSH-Px enzyme (Sigma-Aldrich, St. Louis, MO, USA) was used at concentrations ranging 0–5 units/mL to prepare a standard calibration curve. The results are expressed as units/mg protein.

#### 2.4.2. Measurement of Inflammatory Mediators and Brain-Derived Growth Factor (BDNF)

Inflammatory cytokines, including IL-6 (ab234570) and IL-1β (ab255730), together with BDNF (ab21389) were assessed using ELISA kits (Sigma-Aldrich). All the assessments were performed according to the manufacturer’s instructions. In brief, the total protein of tissue samples was extracted using a lysis buffer containing 50 mM Tris-HCl, pH 7.5, 150 mM NaCl, 1 mM EDTA, 1% NP-40, 0.5% deoxycholic acid, 0.1% SDS, 10 µL/mL protease inhibitor cocktail, and 1 mM PMSF and subjected to a centrifugation at 12,000× *g*, 4 °C, for 15 min. Then, 100 µL of sample aliquots were mixed with 100 µL EIA buffer (provided in the kit), plus 100 µL standard in a 96-well microplate coated with 100 µL biotinylated primary antibodies, incubated for 2 h at 30 °C. At the end of the incubation period, the sample was aspirated and subsequently washed three times with wash buffer. Following this step, 100 µL of streptavidin–horseradish peroxidase conjugate solution was added to each well and incubated for 30 min at 30 °C, then washed again. Then, 100 µL of substrate solution (provided in the kit) was added to each well and incubated for 30 min at 30 °C. Absorbance was recorded at 450 nm using a Biotek Synergy 2 plate reader (BioTek Instruments, Inc., Winooski, VT, USA).

#### 2.4.3. Measurement the Cholinergic and Monoaminergic Functions

In this study, acetylcholinesterase (AChE) was investigated and used as an indirect indicator of cholinergic function. The assessment was performed according to the method of Wattanathorn et al. [43]. In brief, an aliquot of 25 µL of sample was mixed with a reaction mixture consisting of 25 µL of 15 mM ATCI, 75 µL of 3 mM DTNB, and 50 µL of 50 mM Tris-HCL, pH 8.0, containing 0.1% bovine serum albumin (BSA) and incubated at room temperature for 5 min in a 96-well plate. The absorbance was measured at 415 nm. Then, 25 µL of 0.22 U·mL^−1^ of AChE was added and incubated for 5 min at room temperature, with the absorbance measured at 415 nm. Acetylcholinesterase (5–1000 µM) was used as a reference standard. The percentage inhibition was calculated using the following equation:

% inhibition = 1 − (A_sample_/A_control_) × 100,

where A_sample_ is the absorbance of the sample extract and A_control_ is the absorbance of the blank (50% of aqueous methanol in buffer).

To measure the monoaminergic activity, the total monoamine oxidase (MAO), monoamine oxidase type A (MAO-A), and monoamine oxidase type B (MAO-B) activities were monitored. Total MAO was measured using a previous method, for which details are described elsewhere [44,45]. A sample was diluted with distilled water until the final concentrations of 5 to 0.00005 mg/mL were obtained. Then, 40 µL of sample was mixed with 120 µL amino substrate solution (2.5 Mm p-tyramine in potassium phosphate buffer), 40 µL chromogenic solution (1 Mm vanillic acid, 0.5 Mm 4-aminoantipyrine, 4 U/mL peroxidase in potassium phosphate buffer), and 40 µL enzyme mixture obtained from rat brain homogenate. Reactions were observed at 490 nm using a microplate reader (Chromate 4300). Between readings, the plates were incubated at 37 °C. Absorbance readings were performed every 3 min over a period of 42 min.

MAO-A and MAO-B activities were determined using a kynuramine determination assay. Human MAO-A and MAO-B were used as sources of enzyme. The brain homogenate was mixed with a reaction mixture consisting of kynuramine (45 and 30 mΜ for MAO-A and MAO-B), a substrate of MAO-A and MAO-B. Then, they were dissolved in DMSO (0–10 mg/mL of extract). Following this step, 0.0075 mg/mL MAO enzyme in potassium phosphate buffer (100 mM, pH 7.4) was added and incubated at 37 °C for 20 min. At the end of the incubation, 400 μL 2N NaOH and 1000 μL distilled water were added to stop the reaction. In this study, 4-hydroxyquinoline produced from kynuramine induced by MAO-A and MAO-B was measured via fluorescence at λex = 310 nm and λem = 400 nm. The standard curve was prepared from 4-hydroxyquinoline (0.025–2.00 μM) in potassium phosphate buffer (pH 7.4). In brief, 500 μL of each concentration was mixed with 400 μL 2N NaOH and 1000 μL distilled water before measuring the fluorescence [46].

### 2.5. Determination of Lactobacillus *spp*. and Bifidobacterium *spp*.

The feces of each rat were collected via metabolic cages at baseline and every 7 days throughout the study period, followed by their dilution with phosphate buffer (pH 7.4) (1:9 *w*/*v*) and then serial dilution using the same diluent to prepare samples at concentrations ranging between 10^−2^ and 10^−8^. Then, a 100 μL aliquot of each concentration was inoculated, in duplicate, by surface-spreading on De Man–Rogosa–Sharpe (MRS) agar plates (Himedia™ LACTOBACILLUS MRS AGAR, HiMedia Laboratories LLC, Kennett Square, PA, USA) and Bifidobacterium agar (Himedia™). Then, they were incubated at 37 °C for 48 h in a W-Zip standing pouch by placing an anaerobic GasPak™ (MGC, Mitsubishi, Japan) into a W-Zip pouch in order to induce anaerobic conditions. At the end of the incubation, all plates were removed, and *Lactobacillus* spp. and *Bifidobacterium* spp. were separately isolated and counted according to their differing morphologies on culture plates and Gram staining under the microscope. Data are expressed as Colony Forming Units (CFU)/mL.

### 2.6. Histological Procedure and Nissl Staining

The brains were transcardially perfused with a fixative solution containing 4% paraformaldehyde (Sigma-Aldrich, St. Louis, MO, USA) in 0.1 M phosphate buffer with pH 7.4 overnight at 4 °C. Then, they were infiltrated with a 30% sucrose (Merck, Darmstadt, Germany) solution for 48–72 h. Serial sections of frozen tissues were cut on a cryostat (Thermo Scientific™ HM 525 Cryostat, Waltham, MA, USA) to a thickness of 10 µm. All sections were placed onto slides coated with 0.3% aqueous gelatin solution containing 0.05% aluminum potassium sulfate (Sigma-Aldrich, St. Louis, MO, USA). The triplicate coronal sections of brains were stained with 0.25% cresyl violet (Sigma-Aldrich, St. Louis, MO, USA), dehydrated using 2 changes of graded alcohol (70, 95 and 100%) (RCI LabScan, Bangkok, Thailand), placed in xylene (Merck, Darmstadt, Germany), and mounted using DPX mountant (Merck, Darmstadt, Germany). Based on stereotaxic co-ordinates, three representative sections were selected from the rat brain atlas for the medial prefrontal cortex (mPFC) (lateral 5.78 mm, Bregma 1.98) and the hippocampus (lateral 2.74 mm, Bregma −1.06 mm) [47].

Evaluation of neuron density in the prefrontal cortex (layer 2/3 mPFC pyramidal neurons) was performed under an Olympus light microscope model BH-2 (Tokyo, Japan) at 40× magnification. Counting was performed in three adjacent fields, and the mean number was calculated and expressed as the density of neurons per 255 µm^2^.

### 2.7. Statistical Analysis

All the data are shown as the mean ± SEM. Data obtained from repetitive measurement during the behavioral analysis and assessment of *Lactobacillus* and *Bifidobacterium* spp. were analyzed using repeated measures analysis of variance (ANOVA), whereas all biochemical and histological data were analyzed using analysis of variance (ANOVA). Group difference analysis was performed using the Tukey post-hoc test. Differences are considered statistically significant when the *p*-value is less than 0.05.

## 3. Results

### 3.1. Behavioral Assessments

**Figure 3:**
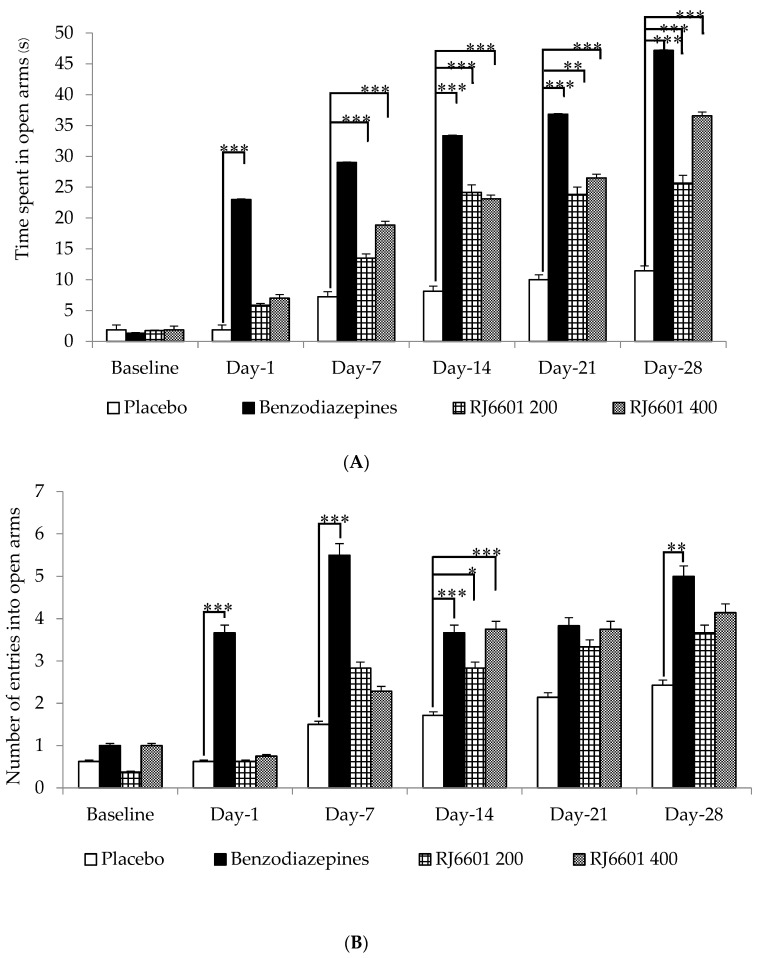
The anxiolytic effect of RJ6601, the polyphenol- and dietary fiber-enriched functional soup, assessed using an elevated plus maze test. Effect of RJ6601 on (**A**) time spent in open arms and (**B**) number of entries into open arms (N = 8/group). Data are presented as mean ± SEM. * *p*-value < 0.05; ** *p*-value < 0.01, *** *p*-value < 0.001; compared to placebo group.

The results for the anxiolytic effect of the RJ6601 soup, which was assessed via an elevated plus maze test, are shown in Figure 3A,B. The data clearly demonstrate that there is no significant difference between groups as observed at baseline or before intervention with RJ6601. The current data reveal that on day 1, benzodiazepine-treated rats spend significantly increased time in open arms, a change that was observed throughout the 28 day study period (*p*-value < 0.001 all; compared to placebo group). Rats that received a high dose of RJ6601, 400 mg/kg BW, showed a significant increase in time spent in open arms on day 7, and this change persisted throughout the study period (*p*-value < 0.001 all; compared to placebo group), whereas a significant change in this parameter was observed in rats treated with RJ6601 on days 14, 21, and 28 of treatment (*p*-value < 0.001, 0.01, and 0.001, respectively; compared to placebo group) as shown in Figure 3A.

Figure 3B shows that, compared to the placebo, benzodiazepine produced a significant elevation in time spent in open arms, but significant changes were observed only on days 1, 7, 14, and 28 (*p*-value < 0.001,0.001, 0.001, and 0.01, respectively; compared to placebo group). A significant increase in this parameter was observed in rats treated with both doses of RJ6601 only on day 14 of treatment (*p*-value < 0.05, and 0.001, respectively; compared to placebo group).

**Figure 4:**
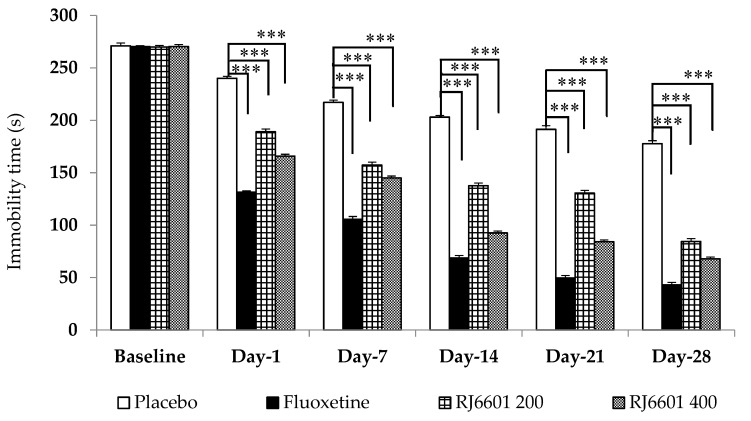
Antidepressant effect of the polyphenol- and dietary fiber-enriched functional soup (RJ6601), assessed using a forced swimming test (N = 8/group). Data are presented as mean ± SEM. *** *p*-value < 0.001; compared to placebo group.

The antidepressant effect of RJ6601 was also evaluated, and the results are shown in Figure 4. Compared to the placebo, fluoxetine and both doses of RJ6601 significantly decreased the immobility time (*p*-value < 0.001, all) in the forced swimming test.

**Figure 5:**
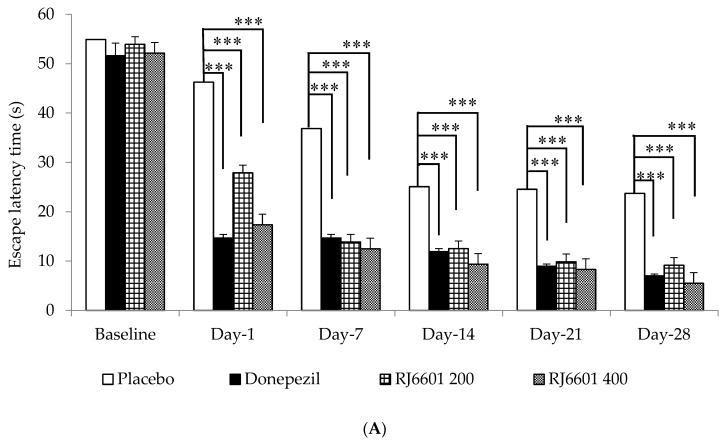

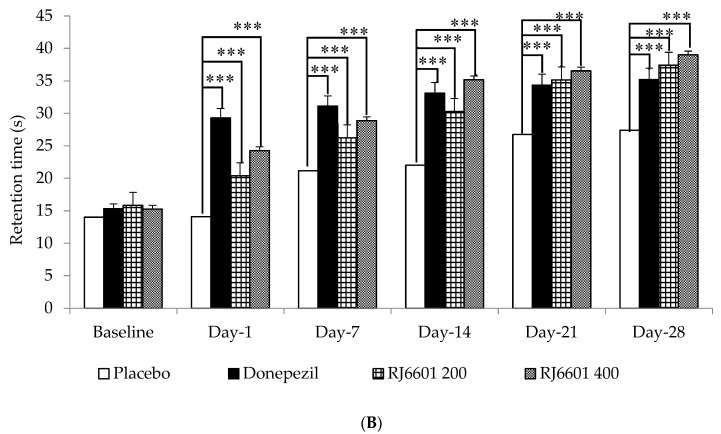
The effect of RJ6601, the polyphenol- and dietary fiber-enriched functional soup, on spatial memory, assessed using a Morris water maze test. Effect of RJ6601 on (**A**) escape latency and (**B**) retention time (N = 8/group). Data are presented as mean ± SEM. *** *p*-value < 0.001; compared to placebo group.

The effect of RJ6601 on spatial memory was assessed using a Morris water maze test, and results are shown in Figure 5A,B. Our results revealed that donepezil and both doses of RJ6601 significantly decreased escape latency but increased retention time (*p*-value < 0.001, all; compared to placebo group).

We also assessed the effect of RJ6601 on nonspatial memory using a novel object recognition test. The discrimination index was assessed after the administration of the tested substances at 30, 60, and 120 min. Table 3 shows that, before the treatments, no significant change in the discrimination index was observed. After a single administration, a significant increase in discrimination index values was observed at 30 and 60 min after administration in rats that received donepezil and both doses of RJ6601 (*p*-value < 0.001, 0.05, and 0.001, and *p*-value < 0.001, 0.01, and 0.001, respectively; compared to placebo group). It was found that on day 7 of treatment, a high dose of RJ6601 increased the discrimination index 30 min after administration, and this change persisted until 60 min after administration (*p*-value < 0.001, and 0.001, respectively; compared to placebo group). At 120 min after administration, rats that received donepezil and RJ6601 at a dose of 200 mg/kg BW showed a significant elevation in their discrimination index (*p*-value < 0.01 and 0.001, respectively; compared to placebo group). No significant change in this parameter was observed at 120 min after the administration of the tested soup. When the treatment was prolonged to 14 days, it was found that all tested substances in this study had significantly increased their discrimination index values at 30, 60, and 120 min after administration (*p*-value < 0.05, 0.05, and 0.001; *p*-value < 0.001, 0.001, and 0.001; *p*-value < 0.01, 0.001, and 0.001, respectively; compared to placebo). Our data also show that when the treatment was extended to 21 and 28 days, an elevation of the discrimination index was observed for all time windows of assessment (all *p*-value < 0.001; compared to placebo group).

### 3.2. Biochemical Changes

#### Neurotransmitter Changes

Although the results demonstrate the positive effects of RJ6601 in modulating anxiety, depression, and memory-enhancing effects, the memory-enhancing effect was revealed to be the most positive, and the ultimate goal of this study was to determine whether there was a memory-enhancing effect as the most important requirement (based on in-depth interviews with the targeted customer group, we assessed the changes in the neurotransmitters of the frontal cortex compared to a placebo and donepezil, a standard drug for treating memory impairment). After 28 days of treatment, donepezil significantly decreased MAO, MAO-A, and MAO-B levels in the frontal cortex (*p*-value < 0.001, 0.01, and 0.001, respectively; compared to placebo). Though donepezil also decreased AChE in this area, it failed to produce a significant change. Compared to the placebo group, both doses of RJ6601 significantly decreased AChE (*p*-value < 0.05 and 0.01), MAO (*p*-value < 0.001, all), MAO-A (*p*-value < 0.001, all), and MAO-B (*p*-value < 0.001, all), as shown in Table 4.

To elucidate the possible underlying mechanisms of RJ6601, we also assessed oxidative stress markers, inflammatory markers, and the brain-derived neurotrophic factor (BDNF) in the frontal cortex. Shown in Table 5 are the effects of the placebo and RJ6601 on the oxidative stress markers, including MDA levels, and the activities of the main antioxidant enzymes, such as SOD, CAT, and GSH-Px, in the frontal cortex. The results show that donepezil increased SOD, CAT, and GSH-Px activities in the frontal cortex (*p*-value < 0.001, 0.01, and 0.05, respectively; compared to placebo), and no significant changes in MDA levels were observed. The low dose of RJ6601 significantly increased GSH-Px activity but decreased MDA levels in the frontal cortex (*p*-value < 0.01 all; compared to placebo group). The high dose of RJ6601 produced a significant increase in CAT and GSH-Px activities but decreased MDA levels (*p*-value < 0.001 all; compared to placebo group) in this area.

**Figure 6:**
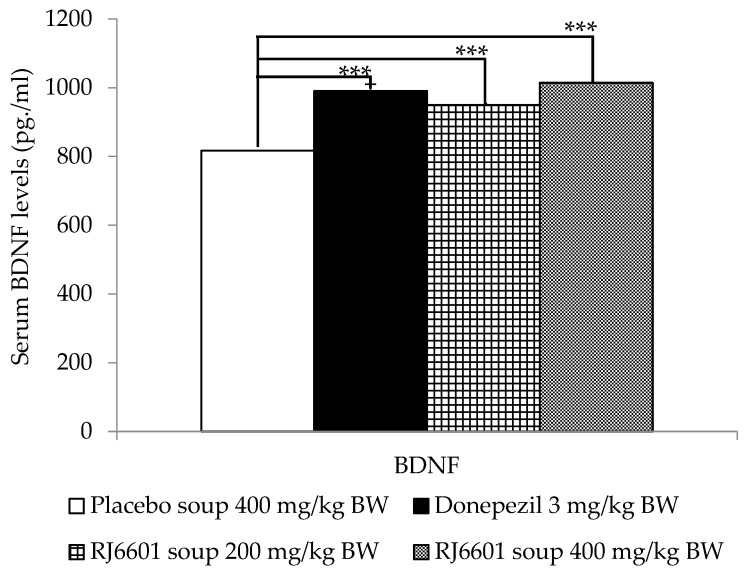
The effect the polyphenol- and dietary fiber-enriched functional soup (RJ6601) on serum brain-derived neurotrophic factor (BDNF) (N = 8/group). Data are presented as mean ± SEM. *** *p*-value < 0.001; compared to placebo group.

Table 6 shows that donepezil and both doses of RJ6601 failed to induce significant changes in either IL-1β or IL-6. However, a significant elevation of BDNF was observed in donepezil and both RJ6601 treatment groups, as seen in Figure 6.

### 3.3. Changes in Lactobacillus *spp*. and Bifidobacterium *spp*.

**Figure 7:**
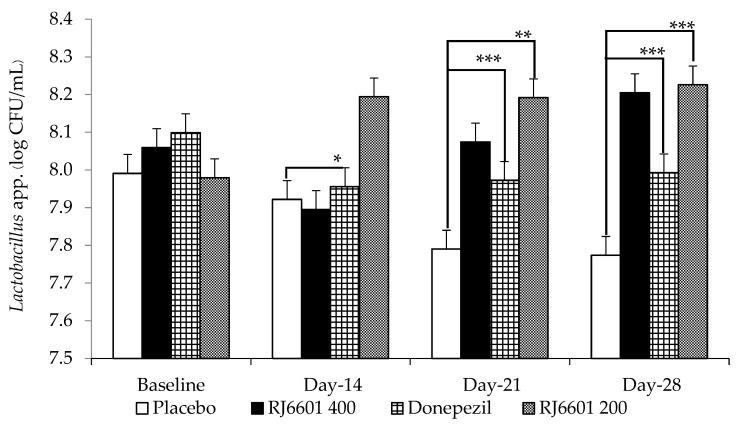
The effect of the polyphenol- and dietary fiber-enriched functional soup (RJ6601) on the density of *Lactobacillus* spp. (N = 8/group). Data are presented as mean ± SEM. * *p*-value < 0.05, ** *p*-value < 0.01, and *** *p*-value < 0.001; compared to placebo group.

**Figure 8:**
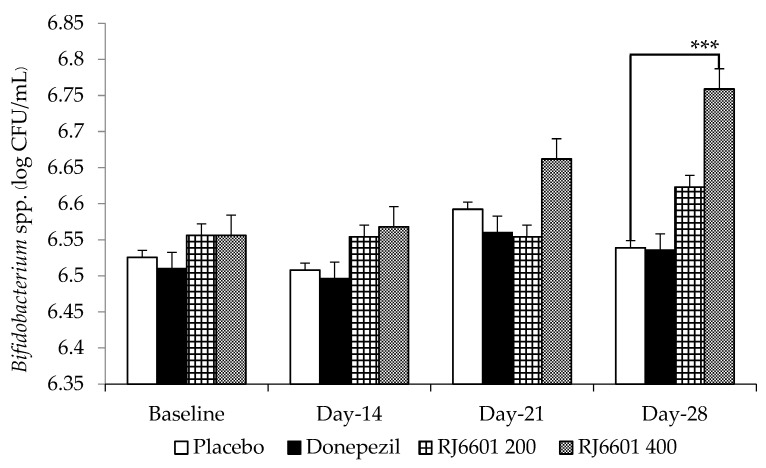
The effect of the polyphenol- and dietary fiber-enriched functional soup (RJ6601) on the density of *Bifidobacterium* spp. (N = 8/group). Data are presented as mean ± SEM. *** *p*-value < 0.001; compared to placebo group.

It was found that donepezil failed to produce a significant change in the density of *Lactobacillus* spp. and *Bifidobacterium* spp. Figure 7 shows that donepezil failed to produce significant changes on the amount of *Lactobacillus* spp., whereas RJ6601 at a dose of 200 mg/kg BW significantly increased the density of *Lactobacillus* spp. at days 14, 21, and 28 of treatment (*p*-value < 0.05, 0.001, and 0.001, respectively; compared to placebo group). However, the high dose of RJ6601 produced a significant increase in this parameter on days 21 and 28 (*p*-value < 0.01 and 0.001, respectively, compared to placebo group). The effect of RJ6601 on the density of *Bifidobacterium* spp. was also investigated, and the results are shown in Figure 8. Only the high dose of RJ6601 increased the density of *Bifidobacterium* spp.

### 3.4. Histological Changes

**Figure 9:**
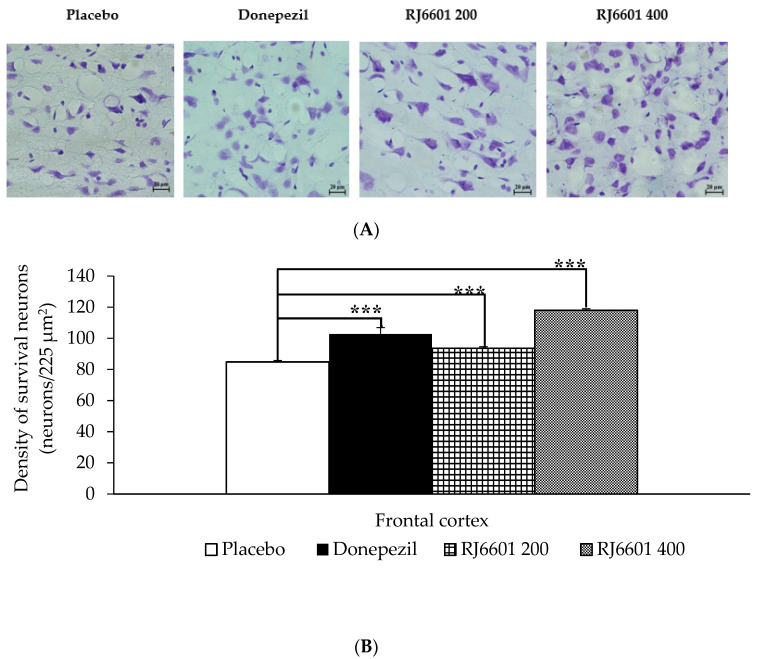
The effect of the polyphenol- and dietary fiber-enriched functional soup (RJ6601) on density of neurons in the frontal cortex. (**A**) Representative histological pictures showing neuron density in the frontal cortex. (**B**) Bar graph comparing neuron density in the frontal cortex of placebo and RJ6601-treated rats. Data are presented as mean ± SEM. *** *p*-value < 0.001; compared to placebo group.

Figure 9A,B demonstrate the effect of RJ6601 on the density of neurons in the frontal cortex. Our results showed that both doses of RJ6601 significantly increased neuron density in this area (*p*-value < 0.001 all; compared to placebo group).

## 4. Discussion

The results of the current study demonstrate that the polyphenol- and dietary fiber-enriched functional soup has anxiolytic, antidepression, and memory-enhancing effects. The functional soup can also enhance BDNF and neuron density in the prefrontal cortex. It also decreases oxidative stress and enhances the cholinergic and monoaminergic systems in this area. The developed functional soup, or RJ6601, also increases the amounts of *Lactobacillus* spp. and *Bifidobacterium* spp. in feces as well as in serum BDNF.

There is accumulating evidence demonstrating that monoamine neurotransmitters such as serotonin (5-HT), norepinephrine (NE), and dopamine (DA) play vital roles in the pathophysiology of mood and memory disorders [48]. The physiological disturbances of 5-HT, NE, and DA, due to either reduced presynaptic release of these neurotransmitters or aberrant signal transductions, give rise to alterations in the regulation or function of receptors and/or impaired intracellular signal processing, which finally lead to the development of mood and/or memory disorders. The suppression of monoamine oxidase (MAO) is reported to produce improvements in the case of mood disorders such as anxiety [49] and depression [50]. These findings also correspond with our results, which show that RJ6601 suppresses MAO, MAO-A, and MAO-B, and increases in the levels of these monoamine transmitters in turn result in an improvement regarding mood disorders. Monoamine depletion in the prefrontal cortex can induce cognitive deficit [51]. In addition to monoamine, acetylcholine (ACh) also plays a critical role in learning and memory. An elevation in central ACh levels induced by the suppression of acetylcholinesterase (AChE) can enhance memory ability and comprehensively improve brain function [52]. Our results are also consistent with the mentioned findings.

It has been found that pyramidal neurons in the prefrontal cortex play an important role in modulating various transmitter systems, including monoamine and cholinergic systems [53]. The survival of neurons in this area is under the influence of BDNF [54], which also plays a role in the pathophysiology of mood disorders and in the mechanism of action of therapeutic agents [55]. The level of BDNF is modulated by both oxidative stress [56] and gut microbiota such as *Lactobacillus* spp. and *Bifidobacterium* spp. [57]. Therefore, we suggest that the improvement in age-related disorders such as anxiety, depression, and memory performance induced by RJ6601, as observed in this study, may be partly associated with a reduction in oxidative stress and the elevation of both *Lactobacillus* spp. and *Bifidobacterium* spp., which in turn increase BDNF, leading to the increased survival of neurons in the prefrontal cortex. This change leads to enhancements in monoaminergic functions that result in improvements related to anxiety, depression, and memory performance. Cholinergic function is also enhanced by the increase in functional neurons in the prefrontal cortex, which in turn enhances memory performance.

RJ6601 can produce improvements related to anxiety, depression-like symptoms, and memory performance, with the highest positive modulation effect being on memory performance. It was clearly demonstrated that rats showed greater improvements in spatial and nonspatial memory when receiving RJ6601 rather than the standard drugs currently being used. In addition, RJ6601, particularly at a high dose, can also improve cholinergic and monoaminergic functions together with oxidative stress status, brain plasticity (reflected by an elevation of BDNF), and functional neuronal circuits in the prefrontal cortex better than the current standard drugs. Our data suggest that RJ6601 is a potential candidate supplement for use in preventing and slowing down age-related memory impairment and neurodegeneration in a convenient and accessible manner. However, confirmation of the observed modulation effects in a clinical trial study is essential.

It has been shown that dietary fiber upregulates *Lactobacillus* spp. and *Bifidobacterium* spp. [58] but decreases oxidative stress [59]. Aside from dietary fiber, polyphenolic compounds and marmelosin can also suppress oxidative stress [60]. Polyphenols also cause increases in both of the aforementioned species [61]. Moreover, our in vitro data (in the Supplementary Materials) also confirm the synergistic interaction among various functional ingredients in RJ6601. Owing to these pieces of information and the high contents of the aforementioned substances in RJ6601, we suggest that the positive modulation effect observed in this study may be partly attributed to the dietary fiber and polyphenolic compounds present in RJ6601 together with the involvement of synergistic interactions between ingredients such as Bael fruit syrup, encapsulated colorant, and banana-derived resistant starch. However, due to the limited budget and thus the number of aged rats available during the study period, it was neither possible to confirm the possible effect of each active ingredient individually nor the interaction between ingredients in vivo. Therefore, determination of the in vivo effects of each ingredient, and their interaction, is also required for further confirmation of the roles of possible active ingredients.

Interestingly, our data are from the first pilot study that clearly reveals that RJ6601, a novel functional soup rich in polyphenols and dietary fiber (according to the composition shown in Table 1, the developed functional soup contains gallic acid, quercetin, and marmelosin at the concentrations of 5 ± 0.000, 15 ± 0.000, and 11 ± 0.00 μg/g of sample powder, respectively, whereas it contains dietary fiber around 8.42 g/100g soup and the content of total polyphenol groups is around 84.42 ± 0.02 μg GAE/mg sample), can enhance both spatial and nonspatial memory performance better than current standard drugs such as donepezil. The data for this supplement suggest that the improvement in transmitters such as ACh and monoamine balance appears to depend principally on the synergistic interaction between various functional ingredients, such as banana-derived resistant starch, Bael fruit syrup, and encapsulated natural colorant. It can also exert a greater influence on the regulation of transmitter balance, oxidative stress status, and brain plasticity. The positive effect in modulating the gut microbiota through *Lactobacillus* spp. and *Bifidobacterium* spp. may in turn play a role in the stimulation of the gut–brain axis, giving rise to elevated BDNF and mitigating brain neurodegeneration, and effects on brain dysfunction may also contribute. However, to address these suggestions, further exploration is still required to precisely understand the underlying mechanisms of action, such as related to the production of short chain fatty acids and the stimulation of the vagus nerve, with clinical trial studies being particularly essential for determining the role of each possible ingredient and the in vivo interactions among various ingredients and their metabolites, before moving forward to its application as a functional food for improving mental wellness.

## 5. Conclusions

The current results demonstrate that the novel functional soup, which is rich in polyphenolic compounds and dietary fiber, represents a potential food innovation that can produce improvements in age-related disorders such as anxiety, depression, and memory performance. The possible underlying mechanisms may partly involve a reduction in oxidative stress and an elevation of BDNF, which in turn enhances functional neuron density in the prefrontal cortex, leading to an improvement in monoaminergic function that results in the alleviation of anxiety and depression. The memory-enhancing effect may occur via the improvement in monoaminergic and cholinergic functions. The positive modulating effect on neurotransmitter balance may be attributed mainly to the interaction of banana-derived resistant starch (which is rich in dietary fiber) and polyphenolic compounds such as gallic acid, quercetin, and marmelosin present in RJ6601. However, we suggest further research in the form of a clinical study to confirm this beneficial effect, with more investigations to facilitate the precise understanding about its possible active ingredients and mechanisms of action.

## Figures and Tables

**Table 1:** The compositions of placebo soup and polyphenol- and dietary fiber-enriched functional soup (RJ6601).

Ingredients	Placebo Soup (%)	RJ6601 Soup (%)
Wheat flour	50.0	25.0
Unripe banana-derived resistant starch	0.0	25.0
Bael fruit syrup	-	4.0
Corn syrup	4.0	-
Rice bran oil	1.3	1.3
Onion	12.0	12.0
Fish bone stock	-	20.0
Salt	0.1	0.1
Water	20.0	-
Potato	3.8	3.8
Unsalted milk	3.2	3.2
Dried fishbones	0.2	0.2
Natural green colorant	-	0.4
Synthetic green colorant	0.4	-
Inulin	5	5
Total	100.0	100.0

**Table 2:** Treatment groups of the experimental rats.

Treatment Group	Treatment
Group I Placebo	Placebo soup
Group II Positive control	Benzodiazepine (5 mg/kg) was used when the anxiolytic activity [33] was evaluated, while fluoxetine (5 mg/kg BW) [34] and donepezil (3 mg/kg BW) [35] were each assessed
Group III RJ6601 200 mg/kg BW	RJ6601 at a dose of 200 mg/kg BW
Group IV RJ6601 400 mg/kg BW	RJ6601 400 mg/kg BW

**Table 3:** Discrimination index values of rats before the treatment and for various periods of treatment with RJ6601, the polyphenol- and dietary fiber-enriched functional soup, regarding nonspatial memory assessed using an object recognition test. Assessment was performed at 30, 60, and 120 min after substance administration (N = 8/group). Data are presented as mean ± SEM. * *p*-value < 0.05, ** *p*-value < 0.01, and *** *p*-value < 0.001; compared to placebo group.

Discrimination Index (DI)																		
Groups	Baseline			Day-1			Day-7			Day-14			Day-21			Day-28		
	30 min	60 min	120 min	30 min	60 min	120 min	30 min	60 min	120 min	30 min	60 min	120 min	30 min	60 min	120 min	30 min	60 min	120 min
Placebo	29.08 ± 4.21	26.65 ± 2.26	26.52 ± 1.72	25.81 ± 3.25	27.47 ± 1.40	38.66 ± 3.26	34.96 ± 2.50	36.67 ± 2.14	36.54 ± 2.50	34.96 ± 2.50	36.67 ± 2.14	36.54 ± 2.50	36.23 ± 3.27	36.18 ± 2.03	36.14 ± 2.14	42.75 ± 3.15	43.84 ± 2.74	42.38 ± 2.34
Donepezil	31.00 ± 5.54	33.19 ± 6.24	26.50 ± 2.45	52.49 ± 5.79 ***	55.31 ± 5.58 ***	44.60 ± 3.78	49.84 ± 6.84 *	57.14 ± 4.86 ***	55.22 ± 2.03 **	49.84 ± 6.84 *	57.14 ± 4.86 ***	55.22 ± 2.03 **	70.82 ± 1.45 ***	61.86 ± 4.30 ***	56.72 ± 3.25 ***	50.85 ± 6.95 ***	57.78 ± 6.12 ***	50.85 ± 4.60 ***
RJ6601 200	32.07 ± 4.34	28.67 ± 3.50	26.60 ± 4.80	40.34 ± 6.41 *	41.74 ± 4.50 **	51.66 ± 6.40	50.17 ± 5.02 *	65.45 ± 4.86 ***	60.53 ± 6.92 ***	60.17 ± 5.02 *	65.45 ± 4.86 ***	60.53 ± 6.92 ***	68.80 ± 1.45 ***	78.25 ± 3.06 ***	82.43 ± 4.09 ***	81.59 ± 2.49 ***	83.11 ± 1.27 ***	91.53 ± 1.96 ***
RJ6601 400	31.92 ± 5.75	26.18 ± 3.92	32.79 ± 3.83	63.14 ± 4.56 ***	53.23 ± 3.56 ***	51.49 ± 4.66	62.35 ± 4.86 ***	62.73 ± 5.10 ***	65.04 ± 3.81 ***	52.35 ± 4.86 ***	62.73 ± 5.10 ***	65.04 ± 3.81 ***	84.46 ± 1.48 ***	86.02 ± 1.69 ***	90.02 ± 0.59 ***	89.15 ± 0.62 ***	90.33 ± 0.82 ***	76.40 ± 6.47 ***

**Table 4:** The effect of RJ6601, the polyphenol- and dietary fiber-enriched functional soup, on acetylcholinesterase (AChE), total monoamine oxidase (MAO, monoamine oxidase type-A (MAO-A), and monoamine oxidase type-B (MAO-B) in the frontal cortex (N = 8/group). Data are presented as mean ± SEM. * *p*-value < 0.05, ** *p*-value < 0.01, and *** *p*-value < 0.001; compared to placebo group.

Frontal Cortex				
Groups	AChE Activity (nmol/mg. Protein)	MAO Activity (µmol/mg. Protein)	MAO-A Activity (µmol/mg. Protein)	MAO-B Activity (µmol/mg. Protein)
Placebo	0.61 ± 0.05	0.14 ± 0.01	0.11 ± 0.01	0.13 ± 0.01
Donepezil	0.57 ± 0.07	0.09 ± 0.02 **	0.07 ± 0.01 ***	0.07 ± 0.01 ***
RJ6601 200	0.47 ± 0.02 *	0.07 ± 0.00 ***	0.06 ± 0.01 ***	0.07 ± 0.01 ***
RJ6601 400	0.42 ± 0.01 **	0.07 ± 0.00 ***	0.04 ± 0.00 ***	0.05 ± 0.00 ***

**Table 5:** Effect of polyphenol- and dietary fiber-enriched functional soup (RJ6601) on the activity of oxidative stress markers MDA, SOD, CAT, and GSH-Px in the frontal cortex (N = 8/group). Data are presented as mean ± SEM. * *p*-value < 0.05, ** *p*-value < 0.01, and *** *p*-value < 0.001; compared to placebo group.

Groups	Frontal Cortex			
	MDA Levels	SOD Activity	CAT Activity	GSH-Px Activity
	(ng/mg Protein)	(Units/mg Protein)	(Units/mg Protein)	(Units/mg Protein)
Placebo	0.28 ± 0.03	13.30 ± 2.39	1.72 ± 0.25	1.21 ± 0.16
Donepezil	0.24 ± 0.03	23.54 ± 3.17 **	5.68 ± 0.84 ***	2.12 ± 0.42 *
RJ6601 200 mg/kg BW	0.17 ± 0.00 **	15.46 ± 0.11	2.97 ± 0.38	2.37 ± 0.15 **
RJ6601 400 mg/kg BW	0.16 ± 0.00 ***	17.03 ± 0.12	4.23 ± 0.17 ***	2.73 ± 0.20 ***

**Table 6:** The effect of RJ6601, the polyphenol- and dietary fiber-enriched functional soup, on inflammatory markers such as IL-1β and IL-6 in the frontal cortex (N = 8/group). Data are presented as mean ± SEM.

Groups	IL-1β (pg/mL)	IL-6 (pg/mL)
Placebo	131.93 ± 3.13	57.50 ± 0.39
Donepezil	128.98 ± 3.11	58.90 ± 1.86
RJ6601 soup 200 mg/kg BW	128.77 ± 3.54	56.39 ± 0.37
RJ6601 soup 400 mg/kg BW	127.04 ± 1.36	55.89 ± 0.09

## Data Availability

The data presented in this study are available on request from the corresponding author. The data are not publicly available due to trade secrecy and the ongoing patent registration process.

## Supplementary Materials

The following supporting information can be downloaded at: https://www.mdpi.com/article/10.3390/foods13142170/s1, Table S1. Biological activities including antioxidant, acetylcholinesterase suppression activity (AChEI), monoamine oxidase suppression activity (MAOI), and cyclo-oxygenase II (COX II) suppression activity of placebo soup and JW6601 soup.
