# Supplementary material for: Anxiolytic, Antidepression, and Memory-Enhancing Effects of the Novel Instant Soup RJ6601 in the Middle-Aged of Female Rats
Source: Foods. 2024 Jul 9;13(14):2170. doi: 10.3390/foods13142170 (PMC11276534; doi:10.3390/foods13142170)
Supplement: Supplementary file 1 [file foods-13-02170-s001.zip › foods-3074145-supplementary.pdf]

Supplementary material 1

Table S1 Biological activities including antioxidant, acetylcholinesterase suppression activity (AChEI), monoamine oxidase suppression activity (MAOI), and cyclo-oxygenase II (COX II) suppression activity of placebo soup and JW6601 soup.

Biological activity EC50 (µg/mL)	Bael syrup	Banana-derived resistant starch	Green natural colorant from the mixture of kale and celery	Placebo	RJ6601
DPPH	56.14±0.33	105.29±0.88	3.19±0.45	48.29±0.05	13.42±0.18
AChEI	21.71±0.38	73.10±0.57	7.76±0.39	43.62±0.03	19.39±0.04
MAOI	34.78±0.31	95.17±0.88	42.63±1.62	17.42±0.02	12.37±0.04
COX-2 inhibitor	11.78±0.69	52.90±0.85	13.54±0.01	118.68	38.97±0.05

According to this table , our data showed that EC50 of antioxidant activity assessed by using DPPH assay revealed that RJ6601showed lower EC50 than bael syrup, banana-derived resistant starch and green natural colorant from the mixture of kale and celery. Furthermore, MAOI also showed the same pattern of change.
